# Inhibition of DNA methylation during chronic obstructive bladder disease (COBD) improves function, pathology and expression

**DOI:** 10.1038/s41598-021-96155-4

**Published:** 2021-08-27

**Authors:** Martin Sidler, K. J. Aitken, Jia-Xin Jiang, Priyank Yadav, Erin Lloyd, Malak Ibrahim, Sanaa Choufani, Rosanna Weksberg, Darius Bägli

**Affiliations:** 1 Paediatric and Neonatal Surgery, Klinikum Stuttgart, Stuttgart, Baden-Württemberg Germany; 2 Developmental and Stem Cell Biology, Research Institute, Hospital for Sick Children, 686 Bay Street, Toronto, ON M5G0A4 Canada; 3 Department of Physiology, Faculty of Medicine, University of Toronto, Toronto, ON Canada; 4 Department of Urology and Renal Transplantation, Sanjay Gandhi Postgraduate Institute of Medical Sciences, New PMSSY Rd, Raibareli Rd, Lucknow, Uttar Pradesh 226014 India; 5 Genetics and Genome Biology, Hospital for Sick Children, 686 Bay Street, Toronto, ON M5G0A4 Canada; 6 Department of Molecular Genetics, University of Toronto, Toronto, ON Canada; 7 Urology Division, Department of Surgery, Hospital for Sick Children, 555 University Avenue, Toronto, ON M5G 1X8 Canada

**Keywords:** Biological techniques, Cell biology, Mechanisms of disease, Bladder, DNA methylation, Epigenetics

## Abstract

Partial bladder outlet obstruction due to prostate hyperplasia or posterior urethral valves, is a widespread cause of urinary dysfunction, patient discomfort and also responsible for immense health care costs. Even after removal or relief of obstruction, the functional and pathologic aspects of obstruction remain as a chronic obstructive bladder disease (COBD). Epigenetic changes, such as DNA methylation, contribute to the persistent character of many chronic diseases, and may be altered in COBD. We tested whether candidate genes and pathways and the pathophysiology of COBD were affected by a hypomethylating agent, decitabine (DAC). COBD was created in female Sprague-Dawley rats by surgical ligation of the urethra for 6 weeks, followed by removal of the suture. Sham ligations were performed by passing the suture behind the urethra. After removal of the obstruction or sham removal, animals were randomized to DAC treatment (1 mg/kg/3-times/week intraperitoneally) or vehicle (normal saline). Bladder function was non-invasively tested using metabolic cages, both one day prior to de-obstruction at 6 weeks and prior to sacrifice at 10 weeks. Residual volume and bladder mass were measured for each bladder. Bladders were examined by immunostaining as well as qPCR. The effects of DNA methyltransferase (DNMT)-3A knockout or overexpression on smooth muscle cell (SMC) function and phenotype were also examined in bladder SMC and ex vivo culture. Residual volumes of the DAC treated group were not significantly different from the NS group. Compared to COBD NS, COBD DAC treatment helped preserve micturition volume with a significant recovery of the voiding efficiency (ratio of the maximum voided volume/maximum bladder capacity) by one third (Fig. 1, p > 0.05). Brain-derived neurotrophic factor (BDNF) variants 1 and 5 were upregulated by COBD and significantly reduced by DAC treatment. Deposition of collagen in the COBD bladder was reduced by DAC, but gross hypertrophy remained. In bladder SMC, DNMT3A overexpression led to a loss of contractile function and phenotype. In bladders, persistently altered by COBD, inhibition of DNA-methylation enhances functional recovery, unlike treatment during partial obstruction, which exacerbates obstructive pathology. The underlying mechanisms may relate to the gene expression changes in BDNF and their effects on signaling in the bladder.

## Introduction

Bladder obstruction in humans is best represented in rodents using the relief of obstruction or chronic obstructive bladder disease (COBD) model, as clinical obstruction most often presents after obstruction has been established^1^. The inciting obstruction can be anatomical or neuronal, and appears in all sexes and ages, though its most common cause is prostate hypertrophy in older males. The societal cost is huge as COBD decreases quality of life, and can lead to incontinence, overactivity, UTI, renal scarring and kidney failure^2–7^. The COBD bladder in vivo shows many stable changes, including de-differentiation and hypertrophy of SMC, as well as gross hypertrophy, loss of voiding function and deposition of extracellular matrix (ECM), which persist for many weeks/months (in animals) and years (in humans) after relief of the obstruction^1,8–13^. The persistent nature of COBD experimentally can be seen in both gene expression, smooth muscle biology and physiology^1,14–18^.

Obstruction initiates mechanical, hypoxic stimuli and ECM remodelling which induce signaling and downstream gene expression changes leading to production of ECM enzymes and release of growth factors^19–22^. A sequence of inflammation, hypertrophy and hyperplasia leads to an almost intractable state which persists despite adequate treatment of the initial obstruction^3,9,23–25^. **During** obstruction, this state is exacerbated by the epigenetic drug, decitabine (DAC)^26^.

While epigenetics works through many mechanisms, DNA methylation is a generally stable form of epigenetic change, which can be targeted pharmacologically and genetically^27,28^. DNA methylation plays a role in gene expression of key obstructive factors, with implications for downstream signaling and pathology^26^. For example, we have seen that DNA methylation alters Brain-derived neurotrophic factor (BDNF) and Connective tissue growth factor (CTGF) during obstruction^26^. We also know that several genes dysregulated during COBD respond to rapamycin^1^, a drug affecting the mammalian target of rapamycin (mTOR) pathway, which can affect DNA accessibility and chromatin^29,30^. Interestingly, DNA methylation itself leads to methylation of mTOR, which correlates positively with altered transcription of the mTOR gene^30^. This is similar to the correlations seen between transcription and DNA methylation in bladder obstruction^26^. While DNA methylation is usually considered to be a repressive factor, the more accurate explanation for the regulation of transcription by methylation is that it affects DNA accessibility. This accessibility will alter binding of both negative and positive regulatory elements^31^. In addition, upstream regulation of a gene by its repressive and activating transcription factors can be altered by methylation of their respective promoters.

To uncover the role of DNA methylation in COBD, we examine the effect of decitabine on bladder physiology, cell biology and cell function. We explore the role of the global DNA methyltransferase (DNMT) inhibitor decitabine during COBD on physiology as well as expression of several genes previously implicated in bladder obstruction^1,26^. Furthermore, we assess overexpression and knockdown of the de novo DNMTs, DNMT3a and 3b, on SMC phenotype.

## Methods

### Animal model

Sprague-Dawley adult female rats were acclimatized for 1 week after arrival, prior to the start of surgeries. Under isoflurane anaesthesia, 24 of the rats weighing 250–280 g underwent obstruction by tying a silk suture around the proximal urethra and a 0.9 mm steel rod, which was then removed leaving the suture in place^1,26,32^. For sham operation, the silk suture was passed behind the urethra without leaving it in place in 12 rats. The sample sizes were derived from our previous work with rapamycin and COBD^1^, as well as DAC and obstruction^26^. After 6 weeks, we recorded micturition patterns during the sleep cycle (subjective night from 6 a.m. to 6 p.m.) of all animals^1^. Next, under anaesthesia, the obstructing ligature was removed from obstructed rats thus creating the COBD model^1^. For shams, the proximal urethra was exposed briefly to control for the de-obstructing surgical procedure at 6 weeks. During the ensuing 4 weeks, half of each group was randomized to either normal saline (NS/vehicle) or the DNA methyltransferase inhibitor, 5-aza-2′-deoxycytidine (DAC, decitabine), at 1 mg/kg 3-times/week intraperitoneally, a dose with proven hypomethylating activity^26^. We started treatment one day after the deobstruction-, or second sham-procedure, as seen in Supplemental Fig. S1. Another 4 weeks after the secondary procedure, we determined the micturition patterns for each mouse again. One day after micturition analysis, animals underwent bladder harvest under Isoflurane anaesthesia, followed by exsanguination. Residual volumes, bladder and body weights were measured at the time of bladder harvesting. Equatorial pieces of bladders for cryosectioning were treated with ice-cold 0.9% sucrose, submerged in optimal cutting temperature compound (OCT), placed on dry ice and then stored at − 70 °C until cryosectioning. Pieces of bladder dome for RNA isolation were treated in RNA later at 4 °C for 24 h, then stored at − 70 °C.

### Micturition analysis

As in other work, we examined micturition using metabolic cages attached to weigh scales and a computer with LoggerPro software^1,26,32^. Since virtually all animals voided during induction of anesthesia, we determined residual volume by direct aspiration of the bladder during organ harvest. The largest voided volume plus the residual volume resulted in the maximum bladder capacity. As a descriptor of how effectively an animal would be able to empty its bladder, we used the ratio of the average voided volume divided by the bladder capacity. At the start of the experiment rats were given a 6-digit number which was utilized for blinding the individuals and samples for analysis. After analysis, the group assignment for each 6-digit number was revealed.

### Study approvals

For these experiments, the Animal Use Protocol was approved by Hospital for Sick Children’s Animal Care Committee, following policies established in the Canadian Council on Animal Care Guide to the Care and Use of Experimental Animals. The study was carried out in compliance with ARRIVE guidelines, and all methods were carried out in accordance with relevant guidelines and regulations.

### Cell culture and overexpression of DNMT3A in human bladder smooth muscle cells

Human bladder SMC were purchased from ScienCell (Carlsbad, California), which established the primary cells in accordance with all appropriate ethical standards. Bladder SMC were maintained in SMC Media (ScienCell) and transferred to starvation media (EMEM plus 0.5% fetal calf serum, Wisent) after plating cells for experiments. The plasmid pcDNA3/Myc-DNMT3A was a gift from Arthur Riggs (Addgene plasmid # 35521 ; http://n2t.net/addgene:35521; RRID:Addgene_35521). DNMT3A and green fluorescent protein (GFP, LONZA) or GFP control plasmid alone were overexpressed in SMC by nucleofection, as in Schroeder et al.^1^. SMC were plated on either gelatin-coated 96 well glass-bottom plates or onto collagen gels in 24 well plates, as in Sidler et al.^26^. Gel contraction was performed after allowing the cells to plate down and integrate into the gels for 24 h. Thereafter the gels were released from the walls and examined at 1 and 24 h for contraction of the gels. In parallel cultures, the cells were stained for DNMT3A using anti-rabbit antibodies to confirm overexpression in > 50% of cells. Degree of contraction was quantified by computer morphometry. Gels were photographed and gel surface area (mm^2^) was determined using computer software, Image J. To analyse the gel contraction in Fig. 4B, the area of the gel filling the well is determined without contraction (uncontracted_gel_area) and compared to the area of the well after release of the well (contracted_gel_area). The calculation for % gel contraction = (uncontracted_gel_area − contracted_gel_area)/(uncontracted_gel_area). Assays were run in quadruplicate and results were expressed as mean ± standard deviation of the mean (SEM).

### Immunofluorescent staining

As in previous work^1,26^, bladder cryosections or human bladder SMC were fixed, permeabilized with 0.2% Triton-X 100, blocked in normal donkey serum (Jackson Immunolabs). Primary antibodies were added overnight at 4 °C, and comprised: smooth muscle myosin (Sigma), desmin (abcam), DNMT3A (abcam), dual phospho-extracellular-signal related kinase 1/2 (ERK1/2, Cell Signaling Technologies/CST), phospho-S6 (CST), WW domain-containing transcriptional regulator protein 1 (WWTR1/TAZ), BDNF (Novus). Details of antibodies can be found in Supplemental Table S2. After washing, secondary antibodies were added (Cy3-donkey anti-mouse, Alexa-647-donkey-anti-rabbit, Jackson Immunolabs), washed and nuclei stained with 4′,6-diamidino-2-phenylindole (DAPI, Sigma). Samples are visualized on a spinning confocal microscope and analysed with Volocity software version 6.3 (Perkin Elmer). Cell numbers were calculated by counting the number of DAPI positive cells on Volocity.

### qPCR

RNA was extracted by Trizol and reverse-transcribed with Superscript III and oligo-dT (ThermoFisher), as previously^26^. qPCR was performed on an MJ Research PCR cycler with primers for BDNF, CTGF and reference genes described^1,26^ or for other genes in Supplemental Table S1 (IDT).

### Cre-induced downregulation of DNMT3a/3b in the ex vivo cultured bladder

Adeno-associated virus (AAV) serotypes were tested for tropism to the bladder SMC and SMC in the mouse bladder using the serotype selection kit (Vigene, Supplemental Fig. S1). AAV6 was selected as tropic for bladder SMC. AAV6-cTNT-cre was produced in HEK293T cells (gift of L. Robinson) and isolated 3 days after transfection of pDGM6 and cTNT-iCre-Tomato, the latter which is targeted to the membrane. pAAV.cTNT.iCre was a gift from William Pu (Addgene plasmid # 69916; http://n2t.net/addgene:69916; RRID:Addgene_69916). TNNT2 (cTNT) expression has been reported in bladder SMC^33,34^, similar to our observations of high levels of TNNT2 in the rat bladder (c(t) values were below 20) and high counts of TNNT2 by RNAseq analysis of mouse bladder (*In Revision*, FASEB). AAV6-cTNT-cre-Tomato was purified using the AAVpro Purification Kit Midi (Takara, Mountainview, California, USA). Briefly, cells were lysed, then the AAV were extracted, treated with nuclease, precipitated and concentrated with an Amicon Ultra-4 filter. Genecopies of AAV were quantified by qPCR, using the AAVpro Titration Kit (for Real Time PCR; Takara), which revealed 2 × 10^9^ GC of AAV6-cTNT-cre/mL. Controls included AAV6-GFP (Vigene) and AAV6-cag-cre-GFP (Vector biolabs). To knockout DNMTs, bladders from DNMT3a/3b-floxed mice^35,36^ were harvested and incubated ex vivo in serum-free media prior to injection of 10 μL AAV (2 × 10^7^ GC) into bladder muscle at three spots using a 28-gauge needle. Bladders were incubated for 2 h in 1 mL of media, at 37 °C, 5% CO_2_, then reconstituted to 5 mL of complete SMCM (ScienCell). Media was changed every 2–3 days. At day 7 post-injection, bladders were frozen in OCT compound for cryosectioning and immunofluorescent staining of DNMT3A, desmin, and smooth muscle myosin, as previously using Alexa-647 rabbit secondaries as above^1,26,32,37,38^. Fluorescence was compared to GFP (AAV6-GFP, or AAV6-CAG-cre-GFP) and Tomato (AAV6-cTNT-cre-Tomato).

### Statistics

Analysis of variance was performed prior to post-hoc t-tests, with p < 0.05 considered significant, on ‘R’. Normal distribution and homogeneity of variances were tested with Shapiro–Wilk’s and Bartlett’s, respectively. If distribution was not normal, a non-parametric Kruskal’s test was utilized. Where variances were unequal, Welch’s t-test was performed. Box and whisker plots indicate the median with quartiles and mean ± SE are presented in the text where differences were noteable.

## Results

### Decitabine (DAC) treatment after relief of obstruction, during COBD, improves bladder voiding efficiency

**Figure 1:**
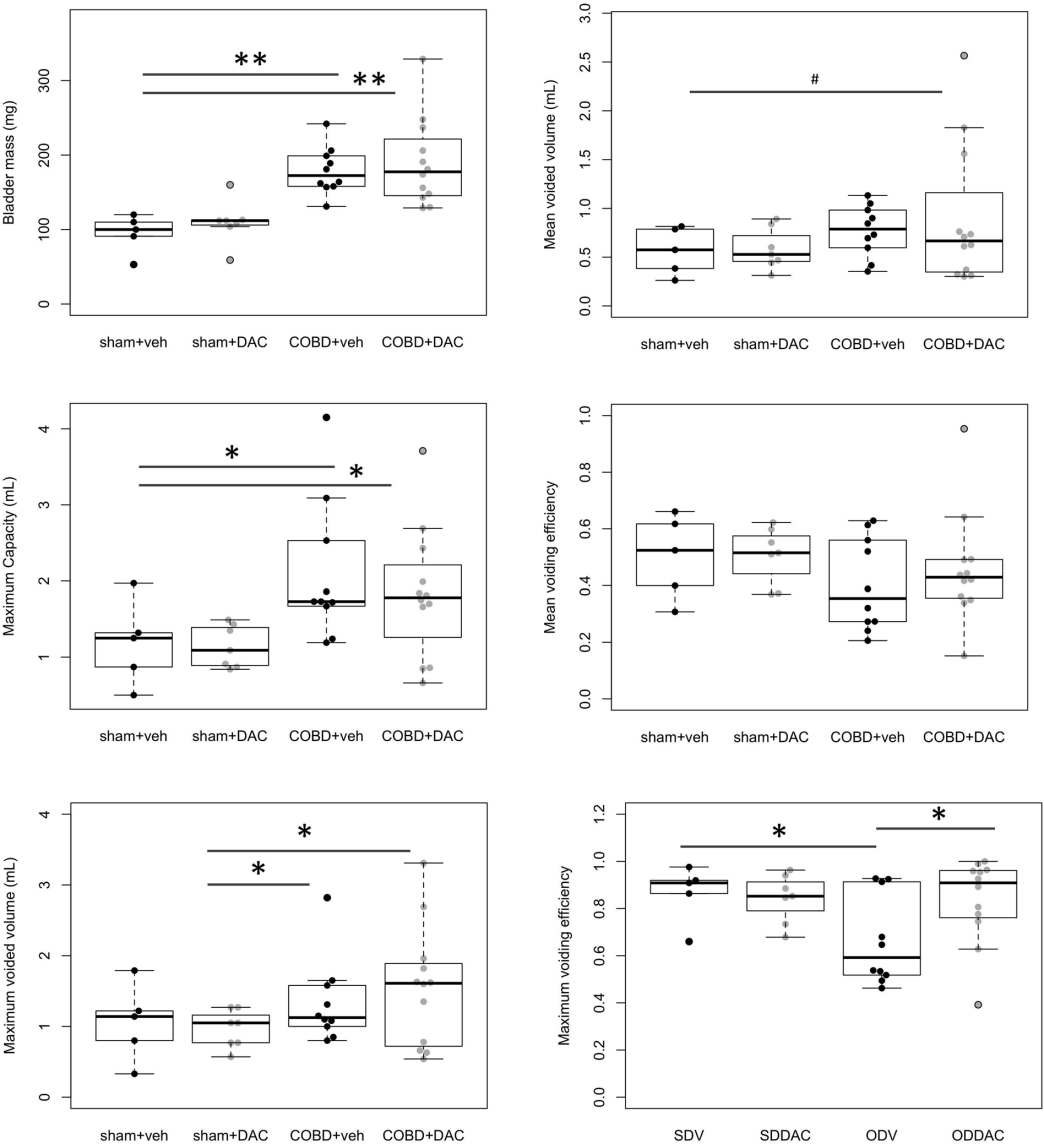
Decitabine treatment improves discrete aspects of bladder physiology and pathology during COBD. Previously we noted that 1 mg/kg/day decitabine (DAC) on physiology during obstruction (PBO) exacerbated the bladder dysfunction^26^. Here we examined DAC treatment in chronic obstructive bladder disease after de-obstruction (COBD). DAC treatment of COBD had either an improvement ( maximum voiding efficiency), no change (**bladder mass, maximum capacity, mean voided volume**) or a modest exacerbation in pathophysiology (maximum voided volume) , ^# ^p < 0.05, One-tailed t-test. *Two-tailed t-test, p < 0.05. **Two-tailed t-test, p < 0.01.

**Figure 2:**
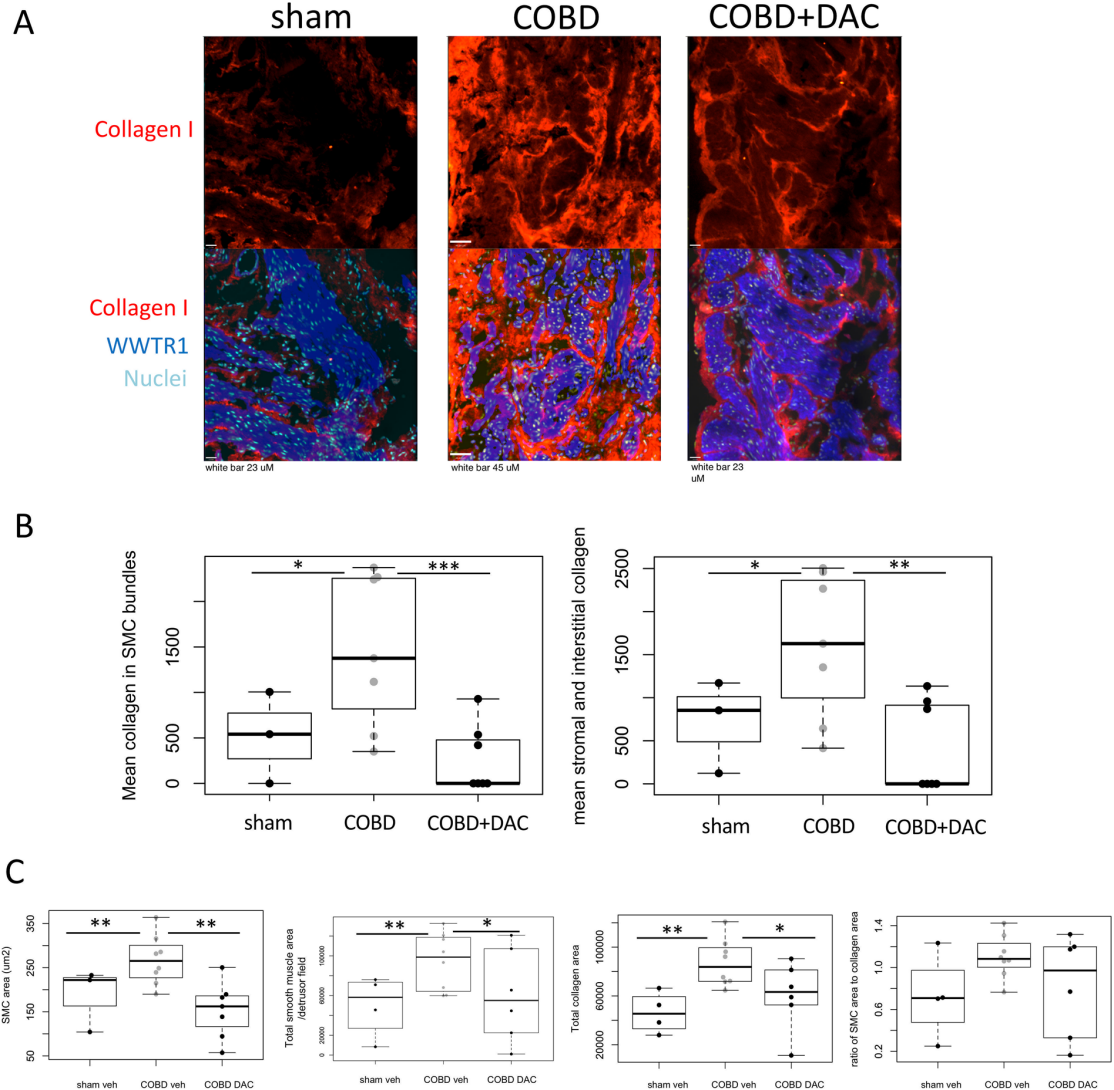
COBD increased collagen type I expression within SMC bundles, while decitabine (DAC) treatment reversed changes in ECM. (**A**) Collagen type I expression by immunofluorescent staining (RED) was increased and more widely distributed during COBD. (**B**) Analysis on Volocity 6.3 revealed that DAC treatment decreased collagen expression significantly in bladder SMC compartments (identified by WWTR1 staining in far-red-blue). (**C**) The SMC area (size of smooth muscle cells) and area occupied by the SMC in the detrusor fields was quantified on Volocity, by counting nuclei and the areas of the detrusor. The SMC area and the total smooth muscle/field was increased by chronic obstruction and reduced by decitabine treatment. Total collagen area was also significantly altered by COBD and DAC treatment. A ratio of the total SMC area to collagen areas in each field were calculated as well (right-most panel), but the ratio was not significantly altered. Nuclei are identified with DAPI (light blue). *p < 0.05 by 1-tailed t-test, **p < 0.05 by 2-tailed t-test, ***p < 0.01 by 2-tailed t-test.

We previously found that DAC treatment during the more acute phase of obstruction worsens bladder function^26^. Here, during COBD, we found that DAC improved bladder maximum voiding efficiency (COBD, 0.66 ± 0.322 vs. COBD + DAC, 0.84 ± 0.29, two-tailed t-test, p = 0.04161, Fig. 1). Other bladder physiologic endpoints were not significantly affected by DAC (Fig. 1, Supplemental Fig. S3). The mean Bladder mass, an indicator of gross bladder hypertrophy, remained increased with COBD (178.9 ± 0.3 mg) vs. sham (94.8 ± 0.5 mg), though was not affected by COBD + DAC (189.3 ± 0.3 mg), Fig. 1, COBD effect by 2-factor ANOVA, p = 7.85 × 10^–6^. Interestingly, the mean area measurements of individual smooth muscle cells (SMC area, Fig. 2A-C) remained above sham levels (186.3 ± 50.4 μm^2^) in COBD (267.4 ± 21.2 μm^2^), but returned to low levels with COBD + DAC (153.5 ± 26.1 μm^2^). The mean of total smooth muscle tissue area/field was increased in COBD (94,487 ± 10,884 μm^2^) vs. sham (50,200 ± 17,874 μm^2^, p < 0.05). Though the individual SMC areas were affected by DAC treatment, the total smooth muscle area/field for COBD + DAC (60,264 ± 21,064 μm^2^) was not significantly different than sham or COBD.

Histologically, detrusor collagen deposition was increased in the interstitial/stromal and endomysial regions during COBD vs. sham (Fig. 2A–C, interstitial collagen in sham = 716 ± 380 vs. COBD = 1610 ± 349; SMC bundle collagen in sham = 515 ± 356 vs. COBD = 1464 ± 347, p < 0.05, 1-tailed t-test). The interstitial and endomysial collagen in COBD was reduced by DAC treatment (COBD + DAC interstitial: 423 ± 218, p = 0.05, endomysial: 269 ± 151, p = 0.005, 2-tailed t-test vs. COBD, Fig. 2B). As both collagen and SMC areas were altered by COBD or DAC, we also looked at the ratio of smooth muscle tissue to total collagen area. While this ratio was increased in COBD, it did not differ between sham or COBD with COBD DAC (Fig. 2C).

### DAC treatment of COBD results in reduction of specific BDNF isoforms

**Figure 3:**
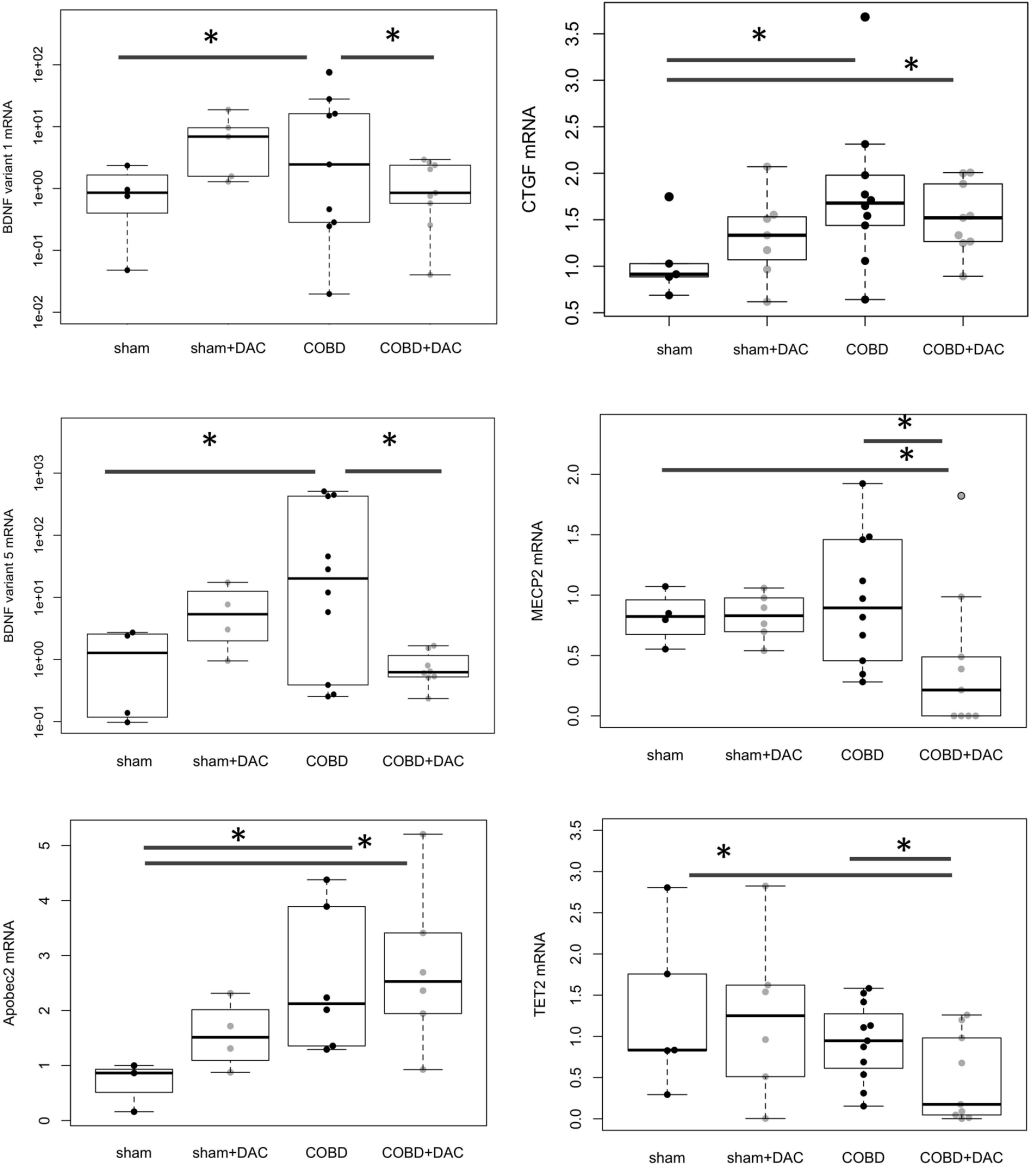
Expression of obstruction- and DAC-responsive genes is altered during COBD. qPCR was performed in COBD bladder tissues to determine expression of genes which we previously found to be restricted by DNA methylation **during** obstruction^26^ in contrast to **after** de-obstruction. BDNF variants 1 and 5 (exons VI and IV) shown here increased significantly with COBD and decreased with DAC treatment in COBD. CTGF was increased with COBD and remained upregulated despite DAC treatment. MECP2, which is a regulator of BDNF, was significantly downregulated with DAC, though during COBD it only tended towards upregulation. We also examined genes regulating DNA de-methylation, APOBEC2 and TET2. APOBEC2 was upregulated with COBD and COBD + DAC. TET2 showed a downregulation with COBD and DAC treatment vs. either sham or COBD alone. Expression of other genes implicated in COBD in the bladder were also examined in Supplemental Fig. S4, but none of these genes showed expression patterns concordant with BDNF mRNA expression. *p < 0.05.

Previously, a high throughput quantitative PCR (HT-qPCR) array showed that BDNF and CTGF increase with 6 week obstruction and correlate with function^26^. In addition, acute obstruction plus DAC treatment, further increases BDNF^26^. Here we found that during COBD, CTGF expression remained higher than sham with or without DAC treatment (Fig. 3, p < 0.05, 2-tailed t-test, sham 0.87 ± 0.08 vs. COBD 1.78 ± 0.27 and COBD + DAC 1.52 ± 0.13). Also, BDNF variants 1 and 5 were both significantly increased (Fig. 3, p < 0.05) during COBD (sham BDNF var1 1.03 ± 0.55 and var5 1.34 ± 0.82, vs. COBD BDNF var1 15.32 ± 8.70, BDNF var5 147 ± 72, respectively). For comparison of sham vs. COBD, negative ddc(t) values were utilized for 1 and 2 tailed t-tests for variants 5 and 1, respectively. COBD levels were reduced by DAC to sham levels (COBD DAC BDNF, var1 1.38 ± 0.39 and var5, 0.81 ± 0.19, p < 0.05, 2-tailed t-test). From our previous work, we also examined genes affected by either COBD or DAC, including Potassium Voltage-Gated Channel Subfamily B Member 2 (KCNB2) and the core clock gene CRY2 (Cryptochrome Circadian Regulator 2) (Supplemental Fig. S4) and Clock^1,26^ (data not shown) as well as epigenetically important genes that might regulate BDNF, e.g. Methyl CpG Binding Protein 2 (MeCP2), Ten-Eleven-Translocation methylcytosine dioxygenase (TET)-1, 2 and 3, and Apolipoprotein B mRNA Editing Enzyme Catalytic Subunit 2 (APOBEC2). CRY2 and KCNB2 were dysregulated significantly during COBD (Fig. 3 and Fig. S4). However, a response to DAC was only seen with BDNF, MECP2 and TET2 (Fig. 3) and KCNB2 (Supplemental Fig. S4). Previous data also showed that DNMT expression is reduced by DAC during obstruction^26^. Notably the DNA methylation related genes MECP2 and TET2 were down regulated by COBD plus DAC and APOBEC2 remained upregulated. The fine tuning of demethylating and methyltransferase enzymes may allow for dynamic alterations in the expression of BDNF. Interestingly, expression of BDNF variants 1 and 5 was concordant with changes in micturition efficiency and detrusor collagen staining.

### BDNF effects on SMC contractility are dependent on DNMT3A

**Figure 4:**
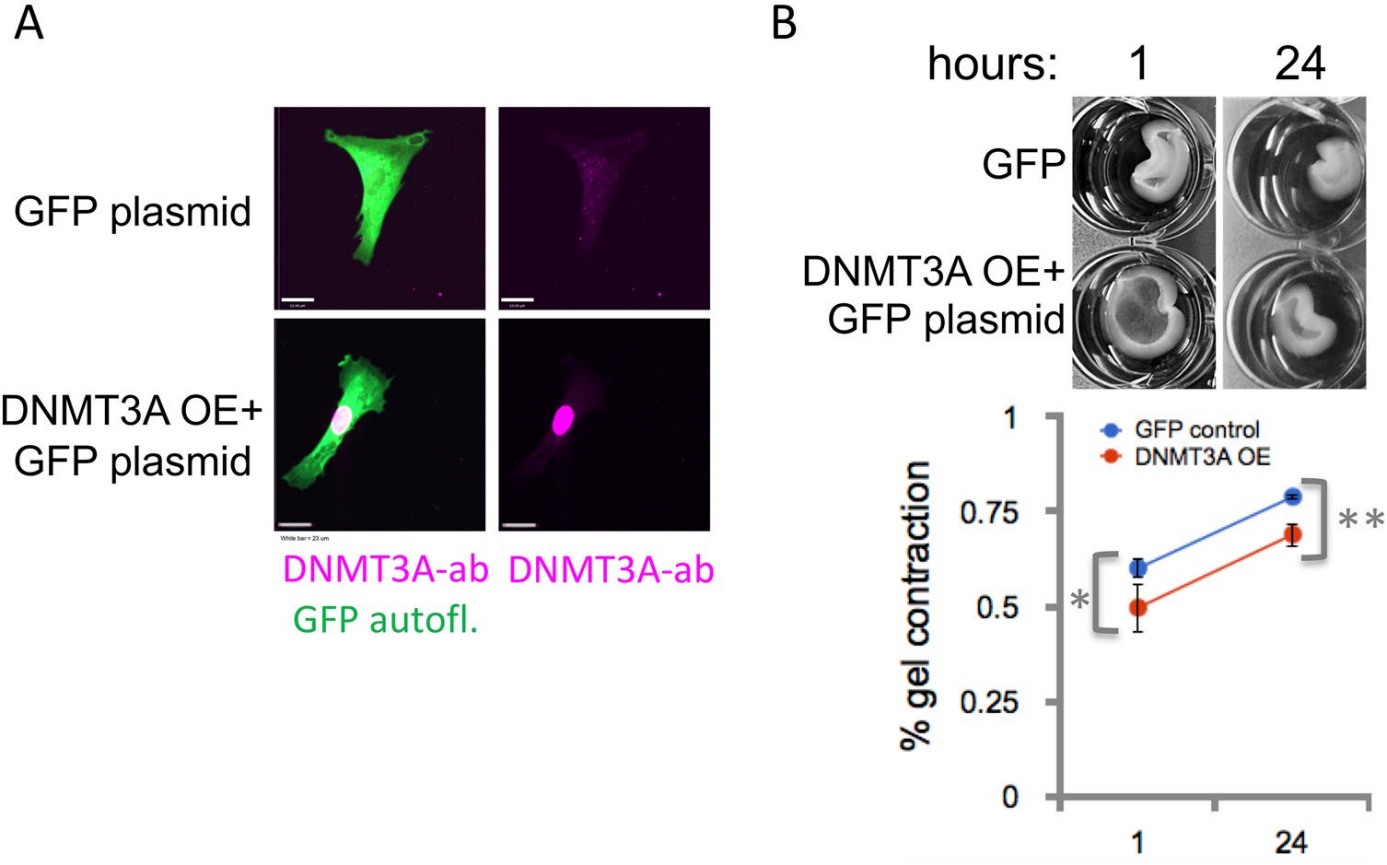
DNMT3A alters contractile function of bladder SMC. (**A**) Overexpression of DNMT3A + GFP (DNMT3A OE + GFP plasmid) vs. GFP plasmid alone was performed by nucleofection in human bladder SMC. Anti-DNMT3A (Magenta-far-red) detected a strong overexpression of DNMT3A in cells with GFP co-expression, representative image shown. All images at same magnification. White bar = 23 microns. (**B**) In parallel cultures, cells were plated onto collagen gels at 3 × 10^4^ cells/mL in 24 well plates and allowed to grow 1 day prior to releasing gels from wells to allow for attachment and contraction. DNMT3A overexpression significantly decreased gel contraction, Overexpression was confirmed in parallel cultures. Representative photomicrographs shown. *p < 0.002.

Previously we found that DNMT3A translocation in bladder SMC was increased in cells plated on abnormal matrix^39^. To determine if DNMT3A overexpression alters SMC function, we examined the DNMT3A in SMC function in vitro*.* Co-expression of DNMT3A with GFP was confirmed in parallel cultures by colocalizing DNMT3A immunofluorescent signal with GFP signal, Fig. 4A. We also confirmed the absence of high levels of DNMT3A expression with GFP plasmid only, Fig. 4A. Importantly, collagen gel contraction was significantly decreased by overexpression of DNMT3A at 1 and 24 h after release of the wells, p < 0.05 (1-tailed) and p < 0.002 (2-tailed), respectively, Fig. 4B.

### Signaling is altered in COBD bladders

**Figure 5:**
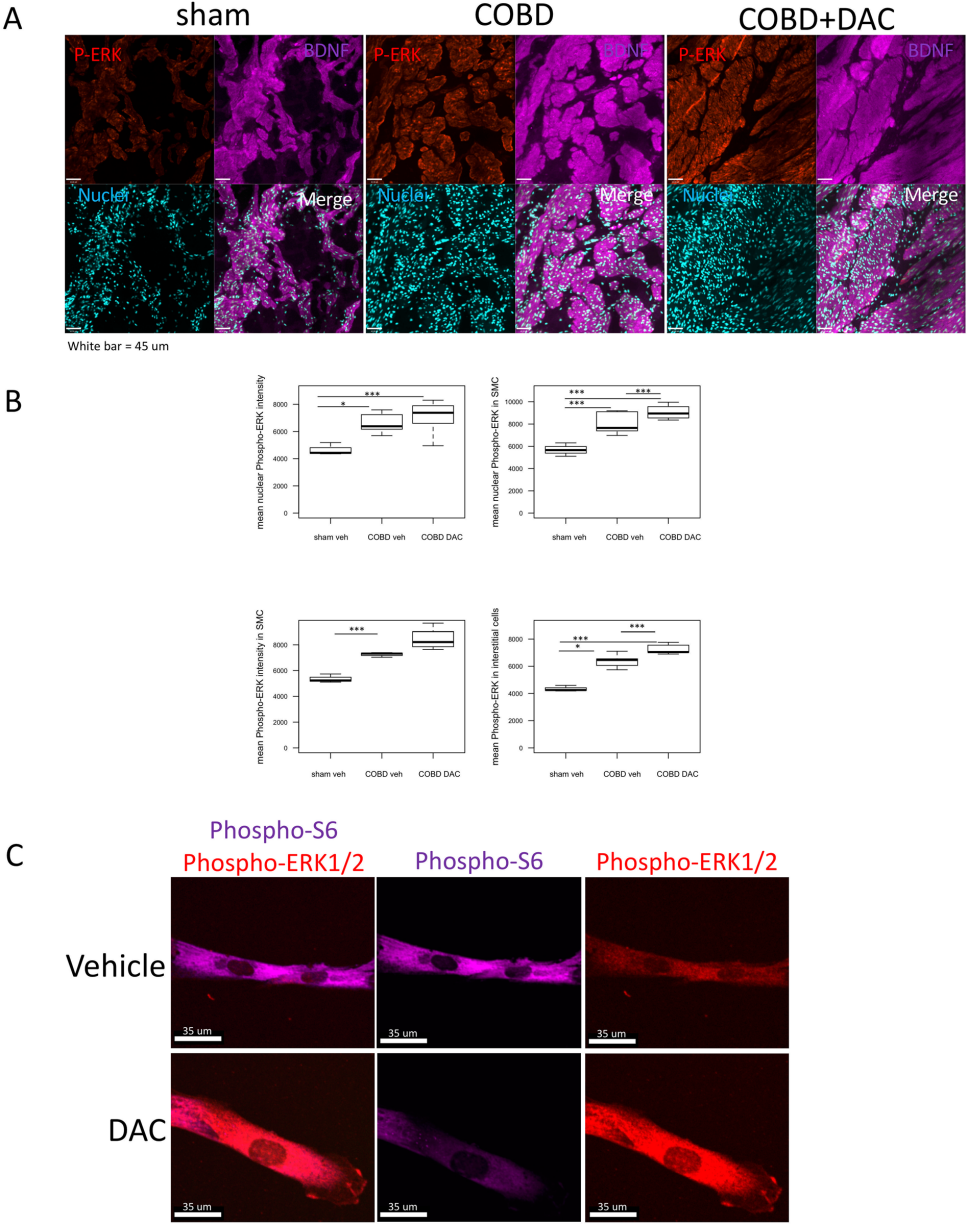
Activation of ERK1/2 is associated with persistent and decitabine (DAC)-resistant features of COBD. (**A**) Localization of activated ERK was performed by quantifying phospho-ERK1/2 (red-Cy3) in the SMC bundles (positive for BDNF, purple-far-red, as in previous work)^26^, and compared to the nuclear staining by DAPI (light blue), on Volocity. (**B**) Staining for Phospho-ERK was most intense in the nucleus of COBD cells. Nuclear phospho-ERK staining of interstitial cells and SMC was increased by DAC treatment. (**C**) Primary human bladder SMC maintained in 2% growth media were treated with vehicle or 2 μM DAC for 48 h to examine if signaling was altered by DAC treatment. Phospho-S6 (purple-Far-red) and phospho-ERK (red-Cy3) were detected by immunofluorescence, which showed a reduction of Phospho-S6 and an increase in phospho-ERK1/2. *p < 0.05, **p < 0.01, ***p < 0.005.

As BDNF increases ERK phosphorylation in bladder smooth muscle cells^26^, we queried here if ERK activity is affected by COBD or DAC treatment. In the SMC compartment, activated ERK was increased in the total cell volumes of the COBD detrusor region. With DAC treatment, phospho-ERK further increased in the nuclei of the detrusor (Fig. 5A,B). To understand if DAC affects key signaling pathways in SMC^38–40^, we inspected ERK and mTOR signaling by immunofluorescent staining in human bladder SMC cultures. DAC increased the intensity of activated ERK in human bladder SMC, yet decreased the intensity of phosphorylated S6 protein, which is a strong downstream indicator of mTOR activity, Fig. 5C.

### Specific loss of de novo DNMTs in situ alters SMC phenotype

**Figure 6:**
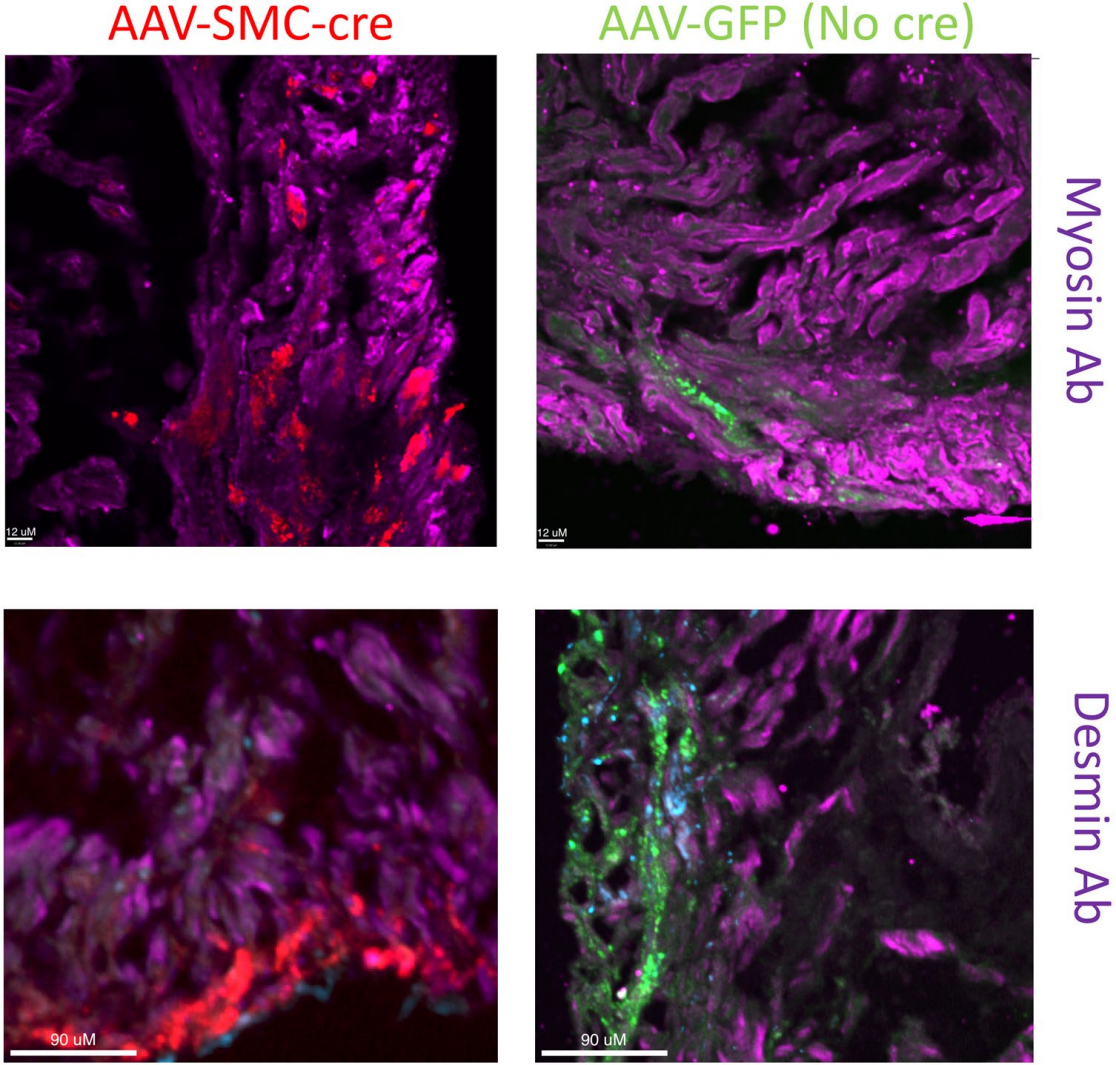
DNMT knockout is associated with altered smooth muscle cell (SMC) phenotype. Bladder SMC targeted AAV6-cre-Tomato (vs. AAV6-GFP) leads to a reduction in DNMT3a/3b (Supplemental Fig. S2). This decrease is associated with a reduction in far-red staining for desmin and smooth muscle myosin (Myh11). Representative photomicrographs shown at the same magnification for each antibody set. White bar for myosin = 12 microns. White bar for desmin staining = 90 microns.

To test the role of DNA methylation on SMC phenotype in situ, we transduced DNMT3a/3b-floxed bladders with AAV-cTNT-cre to create DNMT3a/3b-deficient bladders ex vivo (Supplemental Fig. S1). We detected a modest reduction in expression of both DNMT3A (Supplemental Fig. S1.iv) and the SMC markers desmin and smooth muscle myosin (Fig. 6) in Tomato-positive (DNMT3a/3b deficient) cells as compared to control transduction ex vivo.

## Discussion

After de-obstruction, pathologic sequelae continue in COBD rodent models. This may include frequency, SMC and gross hypertrophy, loss of voiding efficiency and altered bladder capacity, histologic remodeling. In this particular timing of the model, we found that gross hypertrophy, ECM remodeling, loss of voiding efficiency were the main effects on the bladder. Interestingly, we found that collagen deposition as a representation of ECM remodeling and loss of voiding efficiency were recoverable with decitabine (DAC) treatment in vivo. The reduction of contractile function by DNMT3A overexpression could provide a mechanism for the loss of voiding efficiency. The changes in collagen deposition in the smooth muscle regions of our model was consistent with the rescue of collagen gene expression by DNA methylation inhibition in human smooth muscle cells derived from neurogenic bladders^41^, and with bladder ECM remodeling in human COBD^9,42^.

Also consistent with the pathophysiology was the downregulation of COBD-induced BDNF isoforms by decitabine. We know that BDNF has many functions in terms of cell biology of the smooth muscle, as it downregulates higher MW isoforms of smooth muscle myosin and alters differentiation of SMC^26^. This is similar to the effects of DNMT3a and 3b-induced deficiency on myosin and desmin (SMC markers of the bladder) in Fig. 6. Interestingly, desmin has been found to be upregulated during bladder obstruction^43^. We see here interestingly that the negative regulator of BDNF, MECP2 is downregulated by decitabine treatment (Fig. 3). While this would seem inconsistent with BDNF downregulation, MECP2 deficiency by itself can downregulate BDNF^44^ and is part of the complex regulation of BDNF, which includes other positive and negative regulators such as REST corepressor-2 (RCOR2)^45–50^. Interestingly, we found that human bladder SMC rapidly respond to damaged matrix within 2 days by increasing methylation of the RCOR2 CpG island by 7.98%^39^. Consequently, it is interesting to speculate on the role of RCOR2 in regulation of BDNF in bladder SMC. We also note that BDNF, is still highly tropic for the detrusor regions of the bladder (Fig. 5A), similar to BDNF expression during obstruction^26^.

Finally, although remodeling and voiding function are improved in these bladders, gross hypertrophy remains. CTGF, APOBEC2 and KCNB2 expression and ERK-MAP Kinase activity are concordant with this remaining overgrowth and may be playing a role in the continued gross hypertrophy. In prior work, we saw that CTGF associated with hypertrophy of SMC during obstruction^26^. Also, ERK activity is increased during proliferative responses to strain, hypoxia, pressure and ECM in bladder SMC^40,51,52^. These obstructive stimuli can induce bladder SMC proliferation and alter contractile protein expression in an ERK-dependent manner^40,53,54^. Interestingly, ERK activity was increased by DAC both in vivo and in vitro, supporting a role for ERK in the continued hypertrophy of the bladder during DAC treatment of COBD. As COBD relieves the majority of mechanical stress in this particular model with the 6-week obstruction and 4-week de-obstruction timing, mean volumes and residual volumes are reduced on average. In terms of ECM stimuli, the collagen type I deposition associated with COBD regresses with decitabine treatment. However, other ECM changes may continue to be dysregulated, in terms of both expression (collagen type III, elastin, FN1) and activity (MMPs). Collagen type III expression has been found to be upregulated more than collagen type I in clinical and experimental obstruction^55–57^, which could alter the structure of the bladder^22,58–60^ and alter signaling of cells^61,62^. Another major obstructive co-stimulus, hypoxia, tends to emerge during microvascular compression due to wall stress or ECM alterations during obstruction, and these latter stimuli are reduced in COBD. We suggest that persistence of hypertrophy may be governed by epigenetic mechanisms that respond to inhibition of neither DAC nor mTOR^1^. This is consistent with the effects of DAC on mTOR but not ERK signaling in vitro (Fig. 5C). Similarly, the ongoing CTGF expression and ERK activity may be more responsive to agents that promote DNA re-methylation as opposed to de-methylation, or changes in chromatin or non-coding RNAs. Fine tuning the de- and re-methylation of specific gene promoter targets would require an epigenetic CRISPR system targeted with gRNA^63,64^. The effects of decitabine revealed in this COBD model provide a basis for development of a cocktail of targetable DNA methylation treatments and hope for future therapeutic advances.

In summary, the response to epigenetic inhibition differs between obstruction and its more translational model, COBD. While obstruction previously revealed a highly significant increase in gene expression of many genes, including BDNF and CTGF, the response of obstruction to inhibition of DNA methylation worsened the functional and hypertrophic changes in the bladder. This contrasts the effect of DNA methylation inhibition in COBD, where efficiency is improved. DAC treatment of COBD also reverses the upregulation of BDNF, in contrast to the exacerbation of BDNF upregulation by DAC during evolving obstruction^26^. Indeed, the voiding efficiencies of evolving obstruction are also highly significantly decreased by DAC treatment, inversely correlating with the upregulated BDNF expression^26^. This would suggest that the epigenetic context during obstruction and COBD differ, such that, overall, DNA methylation is protective during obstruction, but damaging during COBD. It may be that DAC-induced loss of DNA methylation increases expression of damaging proteins **during obstruction**, such as BDNF, that would normally be constrained. However, **during COBD**, the changes in voiding efficiency, BDNF, collagen type I deposition and signaling, are reversed with DNA methylation inhibition (DAC). This could in part be due to regression of obstructive stimuli during COBD, and altered expression or activity of the epigenetic machinery, such as DNMT3A, MECP2, APOBEC2 and TET2. In contrast to obstruction then, it appears that DAC-induced loss of DNA methylation speeds the proper repair and a return to normal function of the bladder COBD.

## Supplementary Information

Supplementary Information.
