# Supplementary material for: Inhibition of DNA methylation during chronic obstructive bladder disease (COBD) improves function, pathology and expression
Source: Sci Rep. 2021 Aug 27;11:17307. doi: 10.1038/s41598-021-96155-4 (PMC8397724; doi:10.1038/s41598-021-96155-4)

Supplemental Table S1: Primer sequences and conditions for qPCR.

Gene Id	Annealing Temp	Cycle #	5'-3' forward	5'-3' reverse
APOBEC2	63.7	42	IDT PrimeTime® qPCR Primers	IDT PrimeTime® qPCR Primers
BDNF variant 5 (IV)	61	40	AGCTGCCTTGATGTTTACTTTG	CGCCCATGAAAGAAGCAAAC
BDNF variant 1 (VI)	61	40	AATGTGACTCCACTGCCGG	TCACTCTTCTCACCTGGTGGAA
CTGF	58	40	AAGACCTGTGGGATGGGC	TGGTGCAGCCAGAAAGCTC
CRY2	62.4	45	IDT PrimeTime® qPCR Primers	IDT PrimeTime® qPCR Primers
KCNB2	66	40	Qiagen	Qiagen
MECP2	57.5	40	GGGCTCAGGGAGGAAAAGTC	CACGAATGATGGAACGTCGC
TET-1	63	40	GAAACCCTGAATTGGCAAAA	GGGTGAGCTTTCTGATCGAC
TET-2	60	40	CCCTCACTAGAGAAGACAATCGAG	GATCCACTAAACTCCTGACTCTTC
TET-3	63	40	GAGAAGCTAAGCACACCAGAGAAG	CTGCTCATACTGTAGGGGTCAGAG
TNNT2	63.4	40	IDT PrimeTime® qPCR Primers	IDT PrimeTime® qPCR Primers
B2M	57.8	40	CCGTGATCTTTCTGGTGCTTG	AAGTTGGGCTTCCCATTCTCC
HPRT	60.4	40	AGGCCAGACTTTGTTGGATT	GCTTTTCCACTTTCGCTGAT
GAPDH	64.2	40	IDT PrimeTime® qPCR Primers	IDT PrimeTime® qPCR Primers
RPL32	63	40	CATCTGTTTTGCGGCATCA	CACCCTGTTGTCGATGCCTC
SDHA	61.6	40	GGAAGCACACCCTCTCATATG	AGTAGGAACGGATAGCAGGAG

Supplemental Table S2: Antibody concentrations and references.

Antibody (catalogue number, source)	ug/mL or dilution	References
Smooth muscle myosin, Prestige rabbit monoclonal (HPA015310, Millipore Sigma)	0.5 ug/mL	[1]
Desmin (#8592, abcam)	1 ug/mL	[2,3]
DNMT3A (#188470, abcam)	2 ug/mL	[4,5]
dual phospho-ERK1/2 (9106, Cell Signaling Technologies)	2 ug/mL	[6]
phospho-S6 (2211, Cell Signaling Technologies)	2 ug/mL	[7,8]
Collagen Type I (native/helical form of collagen type I , C2456, Sigma-Aldrich)	1:2000	[9]
BDNF (A1307, ABclonal)	1:200	[9,10]
WWTR1 (NB 110-582596, Novus)	1:200	[9,11,12]
Secondary Anti-rabbit, cross-adsorbed against mouse, rat, human, conjugated to Alex-647 (Jackson Immunolabs)	1:400	
Secondary Anti-mouse, cross-adsorbed against mouse, rat, human, conjugated to Cy3 (Jackson Immunolabs)	1:400	

1. Harakalova, M. *et al.* Incomplete segregation of MYH11 variants with thoracic aortic aneurysms and dissections and patent ductus arteriosus. *Eur J Hum Genet* **21**, 487–493 (2013).

2. Tolg, C. *et al.* Differentiation of Skin Derived Stem Cells Into Bladder Smooth Muscle Cells. *Journal of Pediatric Urology* **5**, S20–S21 (2009).

3. Mutlak, Y. E. *et al.* A signaling hub of insulin receptor, dystrophin glycoprotein complex and plakoglobin regulates muscle size. *Nat Commun* **11**, 1381 (2020).

4. Deivendran, S., Marzook, H., Santhoshkumar, T. R., Kumar, R. & Pillai, M. R. Metastasis-associated protein 1 is an upstream regulator of DNMT3a and stimulator of insulin-growth factor binding protein-3 in breast cancer. *Sci Rep-uk* **7**, 44225 (2017).

5. Jiang, J.-X. *et al.* Phenotypic switching induced by damaged matrix is associated with DNA methyltransferase 3A (DNMT3A) activity and nuclear localization in smooth muscle cells (SMC). *PloS one* **8**, e69089 (2013).

6. Aitken, K. J. *et al.* Mechanotransduction of extracellular signal-regulated kinases 1 and 2 mitogen-activated protein kinase activity in smooth muscle is dependent on the extracellular matrix and regulated by matrix metalloproteinases. *Am J Pathology* **169**, 459–470 (2006).

7. Aitken, K. J. *et al.* Mammalian target of rapamycin (mTOR) induces proliferation and de-differentiation responses to three coordinate pathophysiologic stimuli (mechanical strain, hypoxia, and extracellular matrix remodeling) in rat bladder smooth muscle. *Am J Pathology* **176**, 304–319 (2010).

8. Schröder, A., Kirwan, T. P., Jiang, J.-X., Aitken, K. J. & Bägli, D. J. Rapamycin Attenuates Bladder Hypertrophy During Long-Term Outlet Obstruction In Vivo: Tissue, Matrix and Mechanistic Insights. *J Urology* **189**, 2377–2384 (2013).

9. Sidler, M. *et al.* DNA Methylation and the YAP/WWTR1 Pathway Prevent Pathologic Remodeling during Bladder Obstruction by Limiting Expression of BDNF. *Am J Pathology* **188**, (2018).

10. Hang, P. *et al.* Brain-derived neurotrophic factor attenuates doxorubicin-induced cardiac dysfunction through activating Akt signalling in rats. *J Cell Mol Med* **21**, 685–696 (2017).

11. Hwang, J.-H. *et al.* TAZ couples Hippo/Wnt signalling and insulin sensitivity through Irs1 expression. *Nat Commun* **10**, 421 (2019).

12. Li, W. *et al.* ROCK-TAZ signaling axis regulates mechanical tension-induced osteogenic differentiation of rat cranial sagittal suture mesenchymal stem cells. *J Cell Physiol* **235**, 5972–5984 (2020).

Supplemental Figure 1: Animal model schematic. Functional endpoints were collected after 6 weeks of partial bladder outlet obstruction (pre-COBD) or sham (pre-sham) during micturition readings of mice in the metabolic cages. These readings include 12hr total excreted urine volume, micturition fraction, mean voided volume, and functional bladder capacity and maximal single volume voided. Micturition readings collected at 6 weeks were recorded in untreated pre-COBD animals, which were later treated after release with vehicle or DAC (see dark gray boxes) for another 4 weeks. Differences between samples in pre-COBD+veh or pre-COBD+DAC were compared with COBD+veh or COBD+DAC, respectively, to ascertain improvements during the 4 week COBD treatment protocol. These readings were also compared with 10 week sham animals treated with vehicle or DAC in the last 4 weeks. Endpoints collected from harvested bladders after COBD or sham included residual volumes (collected after anaesthesia induction), bladder weights, immunofluorescent staining, histology and RNA expression analysis.

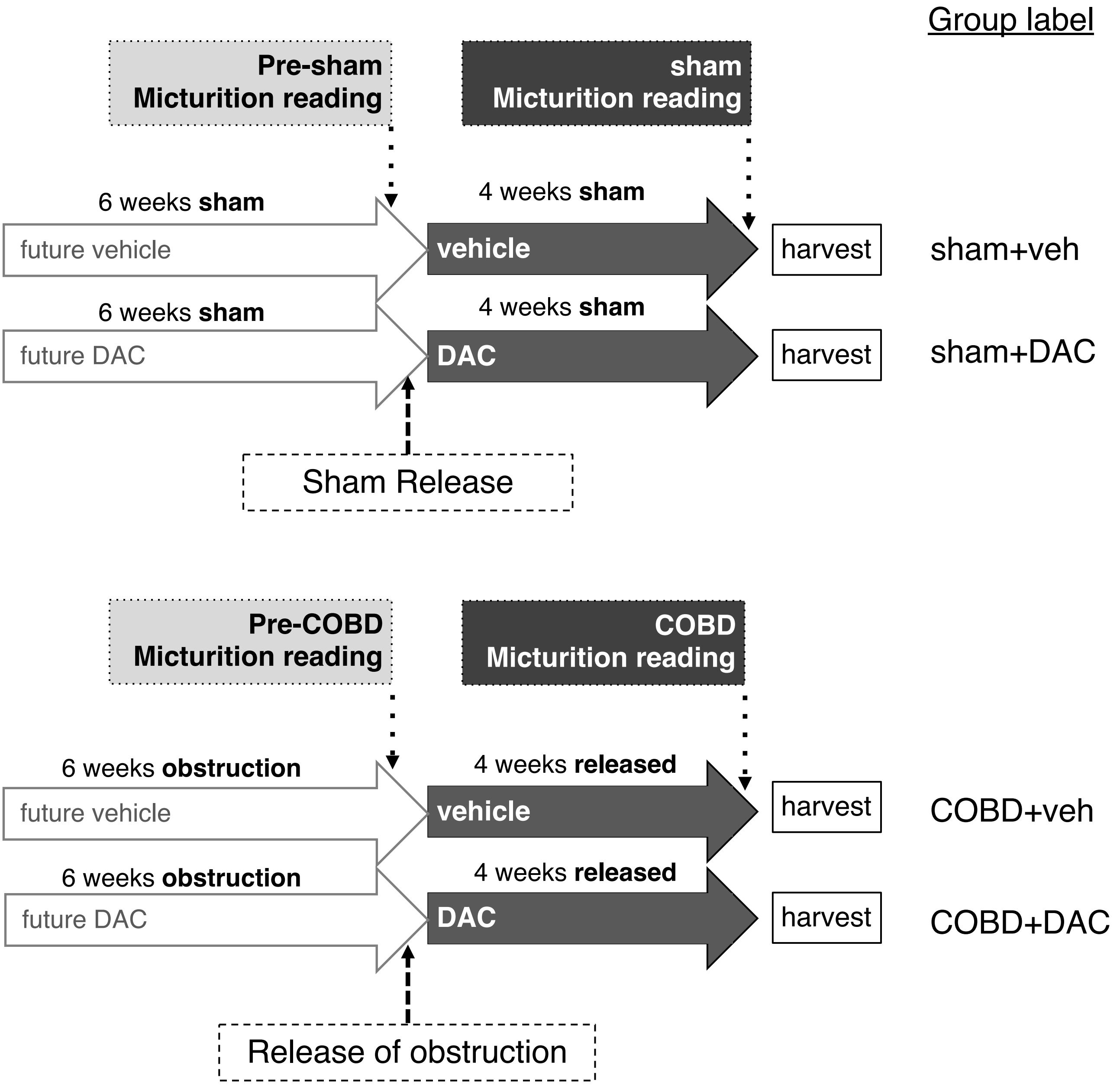

Supplemental Figure S2: Additional micturition parameters (A) from 10w COBD rats did not show large differences. Residual volumes, which were measured after 6 weeks of obstruction or sham plus 4 weeks of de-obstruction or sham, respectively, did not show large differences. Total voided volumes and the total number of micturitions (#micturitions) were also not significantly altered between sham and COBD, although the latter showed a trend in differences between **COBD** and **COBD+DAC**, $p<0.10$ by 2-tailed t-test. (B) at 6 weeks, both obstruction surgeries prior to de-obstruction (PBO) and prior to treatments (future veh or future DAC), were increased above shams, **, p<0.005, by Analysis of Variance. Bladder function of animals randomized to either future vehicle or future DAC treatments did not differ significantly.

A

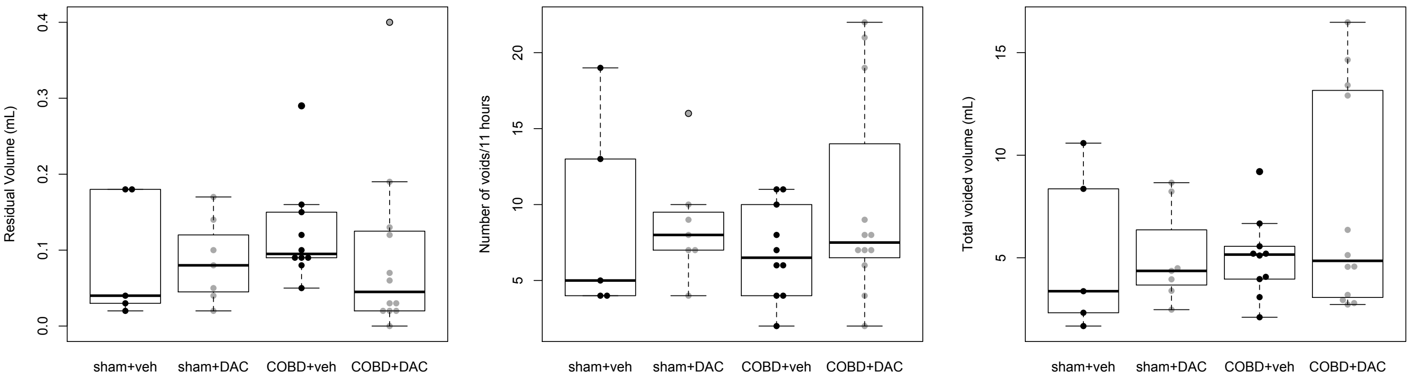

B

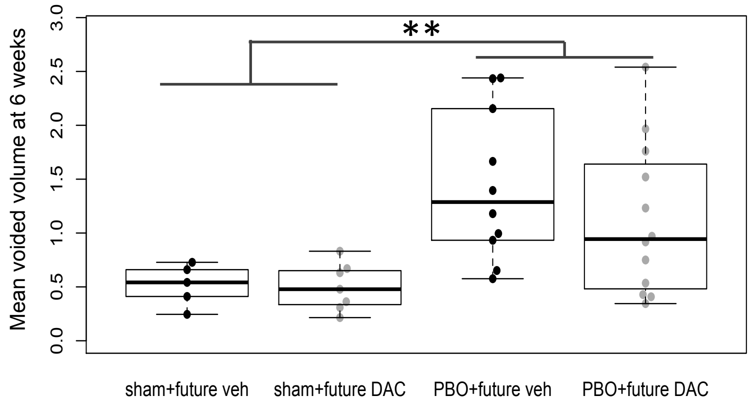

Supplemental Figure S3: AAV transduction methods for bladder SMC. See separate legends in each panel.

i .

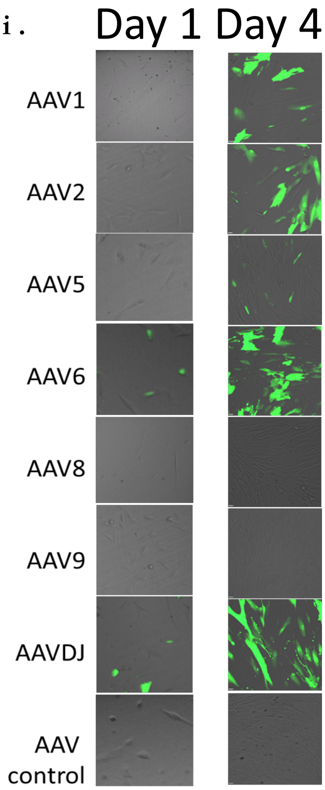

ii .

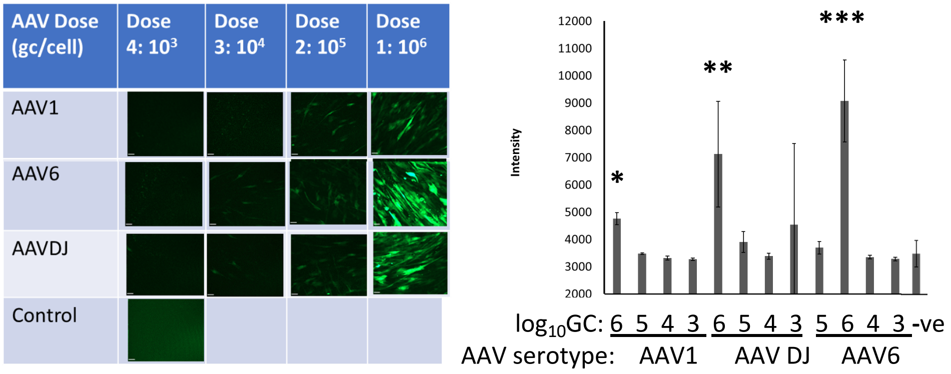

i. AAV transduction by different serotypes by microscopy for GFP fluorescence:(i) Human bladder SMC were transduced with $5X10^5$ genocopies (GC) of AAV-GFP (Vigene) and imaged on days 1 and 4 post transduction. GFP expression was stronger at day 4 for AAV serotypes 1, 2, 6 and DJ. Serotypes AAV6 and DJ continued to increase in GFP expression by day 7 (data not shown). At day 4, the efficiency of AAV6 was 67.4%. Images are merges of GFP fluorescence with light microscopy.

ii. Dose response testing of the most transduced serotypes at day 7: 10^6 GC gave the strongest GFP signal, most significantly with AAV6 and AAVDJ serotypes. 10^5 GC also showed minimal GFP signal. 10^4 and 10^3 had very little GFP signal across all serotypes. This suggests that AAV6 serotype at $1x10^6$ GC/cell is effective for bladder smooth muscle cells. * $p > 0.0005$, ** $p > 10^{-5}$, *** $p > 10^{-10}$ vs. negative control.

iii.

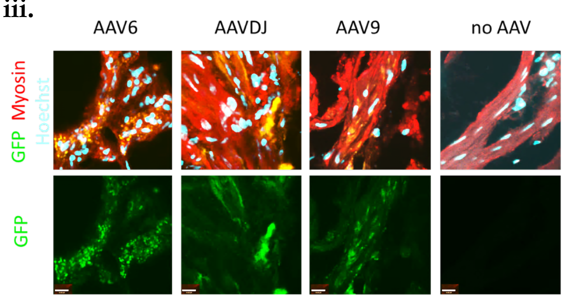

iii. AAV transduction of SMC in the *ex vivo* bladder: Female mice were anesthetized with 2-2.5% isofluorane, and the bladder exposed. The Bladder was removed, the bladder neck sutured, and rinsed with serum-free media. Bladders were transferred from the animal facility to our culture facility, before infecting with 10^{9-11} gc AAV in a BSC hood as per our safety protocols. 10^9 gc AAV did not show high levels of transduction, but 10^{10-11} gc AAV were similar (shown left). Bladders were cultured *ex vivo* serum-free for one week, fixed and embedded into OCT for cryosectioning. AAV-GFP and anti-myosin with anti-rabbit-Cy3 were detected by confocal microscopy. GFP expression in *ex vivo* bladders was significantly lower than in cells *in vitro*. Further experiments that included growth media vs. starvation media showed higher expression for serotype 6 and DJ. *original magnification is 250X.

iv.

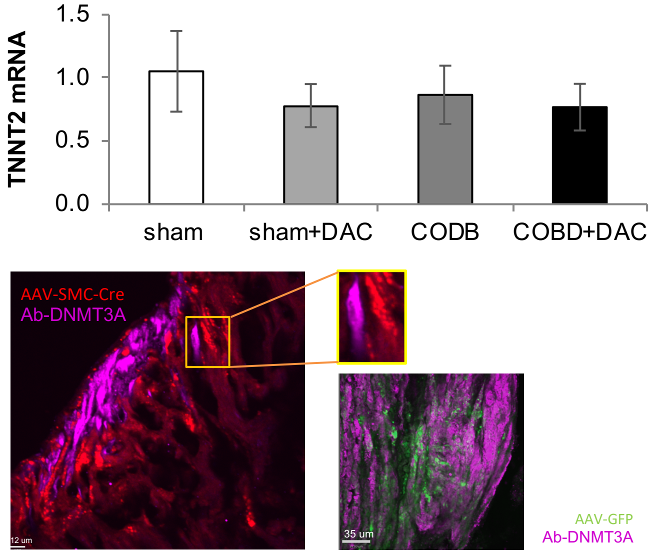

<u>iv</u>: top panel: TNNT2 (cTNT) expression is strongly expressed in the bladder, and not significantly altered by COBD. Ct values for the QPCR were in the range of 15 to 17, indicating strong expression. Bottom panel: Injection of SMC-targeted AAV-cre (RED) into the bladder wall of dnmt3a/3b-floxed mice lead to knockdown of DNMT3A expression (Farred-purple staining). SMC targeting was performed with the cTNT promoter. In contrast AAV6-GFP transduced GFP, but without reducing DNMT3A.

Supplemental Figure S4: (A) Additional qPCR results from sham and COBD bladders reveals alterations in COBD primarily. Expression of obstruction- and DAC-responsive genes is altered during COBD. qPCR was performed in COBD bladder tissues to determine expression of genes, which we previously found to be restricted by DNA methylation during obstruction[26]. **(A) KCNB2** was decreased with COBD, but was not reversed by DAC treatment in COBD. **(B) Cry2** (previously investigated at 6 weeks COBD) was also significantly upregulated during this COBD, but not altered with DAC. These two genes did not show patterns concordant with BDNF mRNA expression from Fig.3. #, $p<0.05$ by 1-tailed t-test of ddc(t) values; *, $p<0.05$ by 2-tailed t-test. **(C)** Quantitative PCR products were electrophoresed on Agarose Gels. Representative products were randomly selected to demonstrate amplification of BDNF isoforms exon IV, VI, CTGF, APOBEC2, and MECP2, TET2 and reference genes RPL32, SDHA and HPRT. With qPCR, the c(t) or threshold of the reaction is the quantitative datapoint, and final amplifications shown here are not quantitative, as (1) reactions may become saturated at the end of a run, and (2) the c(t) values are compared to the average of the reference gene c(t) values.

A

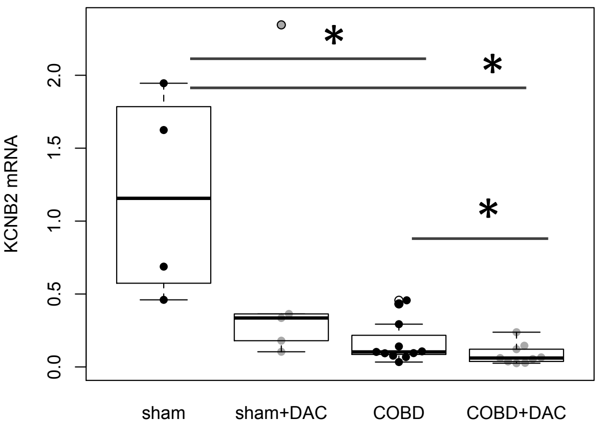

B

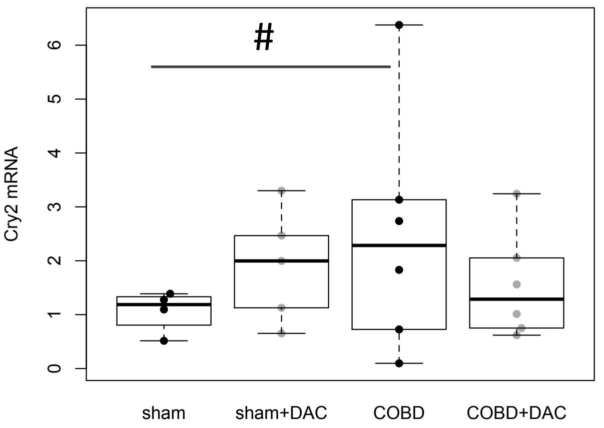

C

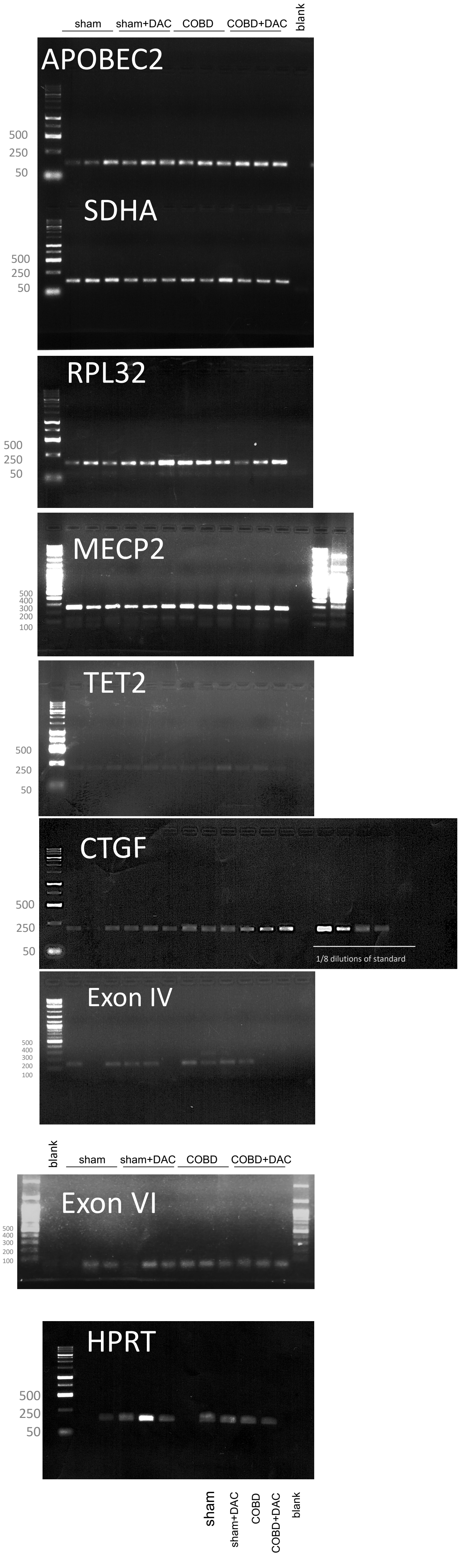

Supplement: Supplementary file 1 — Supplementary Information. [file 41598_2021_96155_MOESM1_ESM.pdf]
